# Competition between Anion-Deficient Oxide and Oxyhydride Phases during the Topochemical Reduction of LaSrCoRuO_6_

**DOI:** 10.1021/acs.inorgchem.4c01568

**Published:** 2024-06-28

**Authors:** Zhilin Liang, Maria Batuk, Fabio Orlandi, Pascal Manuel, Joke Hadermann, Michael A. Hayward

**Affiliations:** † Department of Chemistry, University of Oxford, Inorganic Chemistry Laboratory, South Parks Road, Oxford OX1 3QR, U.K.; ‡ EMAT, University of Antwerp, Groenenborgerlaan 171, Antwerp B-2020, Belgium; § ISIS Facility, Rutherford Appleton Laboratory, Chilton ,Oxon OX11 0QX, U.K.

## Abstract

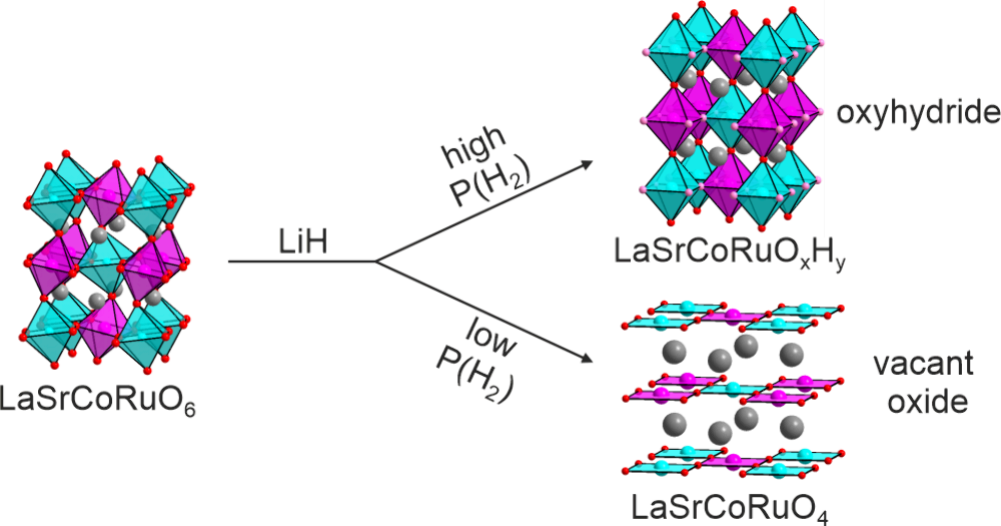

Binary metal hydrides can act as low-temperature reducing agents for complex oxides in the solid state, facilitating the synthesis of anion-deficient oxide or oxyhydride phases. The reaction of LaSrCoRuO_6_, with CaH_2_ in a sealed tube yields the face-centered cubic phase LaSrCoRuO_3.2_H_1.9_. The reaction with LiH under similar conditions converts LaSrCoRuO_6_ to a mixture of tetragonal LaSrCoRuO_4.8_H_1.2_ and cubic LaSrCoRuO_3.3_H_2.13_. The formation of the LaSrCoRuO_*x*_H_*y*_ oxyhydride phases proceeds directly from the parent oxide, with no evidence for anion-deficient LaSrCoRuO_6–*x*_ intermediates, in contrast with many other topochemically synthesized transition-metal oxyhydrides. However, the reaction between LaSrCoRuO_6_ and LiH under flowing argon yields a mixture of LaSrCoRuO_5_ and the infinite layer phase LaSrCoRuO_4_. The change to all-oxide products when reactions are performed under flowing argon is attributed to the lower hydrogen partial pressure under these conditions. The implications for the reaction mechanism of these topochemical transformations is discussed along with the role of the hydrogen partial pressure in oxyhydride synthesis. Magnetization measurements indicate the LaSrCoRuO_*x*_H_*y*_ phases exhibit local moments on Co and Ru centers, which are coupled antiferromagnetically. In contrast, LaSrCoRuO_4_ exhibits ferromagnetic behavior with a Curie temperature above 350 K, which can be rationalized on the basis of superexchange coupling between the Co^1+^ and Ru^2+^ centers.

## Abstract 2

Reaction between LaSrCoRuO_6_ and LiH or CaH_2_ yields LaSrCoRuO_*x*_H_*y*_ oxyhydride phases if the P(H_2_) in the system is sufficient to stabilize these products with respect to hydrogen loss. Conversely, reaction between LaSrCoRuO_6_ and LiH under low P(H_2_) conditions yields the infinite layer phase LaSrCoRuO_4_, indicating that the P(H_2_) of the system can direct the outcome of topochemical reductions.

## Introduction

The refractory nature of binary metal oxides means that the majority of complex transition-metal oxides are prepared at high temperature. These high-temperature “ceramic” synthesis reactions operate under thermodynamic control, resulting in the preparation of the most stable phase, or mixture of phases, for the particular chemical composition reacted. As a result, the range of complex transition-metal oxides, which can be prepared by these routes is quite limited, reflecting the limited number of oxidation states, metal coordination geometries, and three-dimensional packing schemes that are thermodynamically favored under these reaction conditions. Furthermore, there is limited scope to control the products that form for a particular composition, as it is hard to change the thermodynamic balance between product phases, with applied pressure and oxygen partial pressure typically the only parameters which can be used to exert some control.

Topochemical reactions—those that modify the composition of solid compounds by insertion, extraction, or exchange of species while retaining the original structural topology of the phase—can broaden the range of synthesizable solids significantly.^1^ These reactions occur at low temperature, utilizing differences in the mobility of the constituent species in solids to bring about structure-conserving compositional changes. As a consequence, this class of reaction operates under kinetic control, so the products are those phases that form fastest, rather than the phases that are the most thermodynamically stable, thus allowing the preparation of metastable solid compounds. Furthermore, as the identity of the products formed by this class of reaction depends on the relative rates of competing reactions, modification of these reaction rates offers a further way of controlling the products of synthesis in the solid-state.

Binary metal hydrides such as NaH, LiH, or CaH_2_ are effective reagents for low-temperature topochemical anion extraction reactions.^1−3^ For example, NaH can facilitate the topochemical reduction of LaNiO_3_ to the Ni^1+^ phase LaNiO_2_.^4^ Likewise, CaH_2_ can convert SrFeO_3−δ_ to SrFeO_2_^5^ and also be used to prepare a host of other anion-deficient complex oxides containing transition-metal cations in extremely low oxidations states such as Mn^1+^, Co^1+^, Ru^2+^, or Ir^2+^.^6−9^

In addition, binary metal hydrides can also affect anion exchange reactions, allowing for the topochemical synthesis of oxyhydride compounds. Examples include the conversion of LaSrCoO_4_ to LaSrCoO_3_H_0.7_^10^ or the conversion of LaSr_3_NiRuO_8_ to LaSr_3_NiRuO_4_H_4_,^11^ facilitated by reaction with CaH_2_ in both cases.

Here, we describe the reactivity of the double perovskite oxide, LaSrCoRuO_6_, with binary metal hydrides (CaH_2_, LiH) and observe a competition between anion-deficient oxide and oxyhydride product phases, which sheds light on the mechanism of the topochemical processes occurring.

## Experimental Section

### Synthesis of LaSrCoRuO_6_

Samples of LaSrCoRuO_6_ were prepared via a citrate gel method. Suitable stoichiometric ratios of La_2_O_3_ (99.999%), SrCO_3_ (99.99%), Co powder (99.996%), and RuO_2_ (99.99% dried at 800 °C) were dissolved in a minimum quantity of 6 M nitric acid. Three mol equiv of citric acid and 5 mL of analar ethylene glycol were added, and the solution was heated with constant stirring. The gels thus formed were subsequently ground into a fine powder, placed in an alumina crucible, and heated at 1 °C min^–1^ to 900 °C in air. The powders were then reground, pressed into 13 mm pellets, and then heated at 1100 °C in air for 2 periods of 24 h with intermediate regrinding. At the end of the final heating period, samples were cooled at a rate of 5 °C min^–1^ to 450 °C and then removed from the furnace, rapidly transferred to a dry ice-cooled alumina crucible, and allowed to rapidly cool. Synchrotron X-ray powder diffraction data collected from LaSrCoRuO_6_ could be fit by a structural model previously reported for the phase (space group *P*2_1_/*n*) to achieve a good fit as described in the Supporting Information.^12,13^

### Topochemical Reduction of LaSrCoRuO_6_

Samples of LaSrCoRuO_6_ were reduced by the reaction with either LiH or CaH_2_. Samples of LaSrCoRuO_6_ were ground together with 4 mol equivalents of CaH_2_ in an argon filled glovebox. The resulting mixtures were then sealed in evacuated Pyrex ampules and heated as described below. Alternatively, LaSrCoRuO_6_ was ground together with 8 mol equivalents of LiH in an argon filled glovebox. These mixtures were either sealed in evacuated Pyrex ampules and subsequently heated as described below or poured into an open-ended Pyrex tube that was placed within a silica flow-tube, which could be sealed at each end with valves, so that the flow-tube assembly could be inserted into a clam-shell furnace while maintaining an argon atmosphere over the sample mixture. The flow-tube was then purged with argon for 20 min before being heated as described below, under a constant flow of argon, as shown schematically in Figure S8. Reaction progress was monitored by X-ray powder diffraction.

After reaction, samples reduced using LiH were washed with methanol under a nitrogen atmosphere to remove any unreacted LiH and Li_2_O reaction by product. Samples reduced using CaH_2_ were initially washed with a 0.1 M solution of NH_4_Cl in methanol, under a nitrogen atmosphere, to remove any unreacted CaH_2_ or CaO reaction byproduct before being washed with clean methanol.

### Characterization

Reaction progress monitoring and initial structural characterization was performed using laboratory X-ray powder diffraction (PXRD) data collected using a PANalytical X’pert diffractometer incorporating an X’celerator position-sensitive detector (monochromatic Cu Kα1 radiation). Air-sensitive samples were measured in enclosed cells sealed under argon. High-resolution synchrotron powder X-ray diffraction (SXRD) data were collected using the I11 instrument at the Diamond Light Source Ltd. Diffraction patterns were collected by using Si-calibrated X-rays with an approximate wavelength of 0.825 Å from samples sealed in 0.3 mm diameter borosilicate glass capillaries.

Neutron powder diffraction (NPD) data were collected using the WISH diffractometer at the ISIS neutron source from samples contained within vanadium cans sealed under an inert atmosphere. Rietveld refinement of powder diffraction data was performed using the TOPAS Academic software package (V6).^14^

Thermogravimetric analysis (TGA) measurements were performed by heating powder samples under flowing oxygen or nitrogen using a Mettler-Toledo MX1 thermogravimetric microbalance or a PerkinElmer microbalance with exhaust gases monitored by a Hiden Analytical Mass spectrometer. DC magnetization data were collected using a Quantum Design MPMS SQUID magnetometer from samples contained in gelatin capsules. Three-dimensional electron diffraction (3D ED) data were acquired with a FEI Titan 80–300 “cubed” microscope operated at 300 kV. The specimens for the TEM study were prepared by grinding the material in ethanol and depositing a few drops of the suspension onto a copper TEM grid covered by a continuous carbon layer. The specimens were prepared in an Ar-filled glovebox.

## Results

### Topochemical Reactivity of LaSrCoRuO_6_

LaSrCoRuO_6_/CaH_2_ mixtures were heated in sealed Pyrex ampules in the temperature range 330 < *T*/ °C < 380 for 8 day periods. No reaction was observed at temperatures below 350 °C. At temperatures greater than 370 °C, the LaSrCoRuO_6_ perovskite phase decomposed to form mixtures of La_2_O_3_, SrO, Co, Ru, and CaO/CaH_2_. However, in the range 350 < *T*/ °C < 370, PXRD data revealed a new cubic phase had formed (*a* = 7.67 Å), with reflection conditions consistent with the *Fm*-3*m* (#225) space group. A sample for detailed analysis, henceforth referred to as “sample A”, was prepared by heating ∼1.5 g of LaSrCoRuO_6_ with 4 mol equivalents of CaH_2_ within sealed, cylindrical Pyrex ampules (internal diameter 10 mm, lengths 15–20 mm) at 360 °C for 3 periods of 8 days with intermediate regrinding, prior to washing to remove the calcium phases.

LaSrCoRuO_6_/LiH mixtures were heated in sealed Pyrex ampules in the temperature range 300 < *T*/ °C < 340 for 8 day periods. No reaction was observed at temperatures below 310 °C. At temperatures greater than 330 °C, the LaSrCoRuO_6_ perovskite phase decomposed. However, in the range 310 < *T*/ °C < 330, PXRD data revealed that a new, apparently tetragonal phase, had formed (*a* = 5.43 Å, *c* = 7.98 Å), with reflection conditions consistent with body-centering. A sample for detailed analysis, henceforth referred to as “sample B”, was prepared by heating ∼1.5 g of LaSrCoRuO_6_ with 8 mol equivalents of LiH CaH_2_ within sealed, cylindrical Pyrex ampules (internal diameter 10 mm, lengths 15–20 mm) at 320 °C for 3 periods of 8 days with intermediate regrinding, prior to washing to remove the lithium phases.

Analysis, detailed below, revealed that sample B is a mixture of LaSrCoRuO_6–*x*_H_*y*_ oxyhydride phases. To investigate the role of the H_2_ partial pressure (P(H_2_)) generated during the reaction, LaSrCoRuO_6_/LiH mixtures were heated under flowing argon (to minimize the H_2_ partial pressure) in the temperature range 300 < *T*/ °C < 390. No reaction was observed at temperatures below 335 °C, and sample decomposition was observed above 380 °C. However, in the temperature range 335 < *T*/°C < 360, PXRD data revealed the formation of two distinct phases, LaSrCoRuO_5_ (prepared previously by reduction of LaSrCoRuO_6_ with Zr)^15^ and a tetragonal phase (*a* = 5.67 Å, *c* = 6.88 Å), which is identified as LaSrCoRuO_4_, as described below. On raising the reaction temperature to 375 °C, LaSrCoRuO_4_ became the majority phase, but it was not possible to prepare a single-phase sample of LaSrCoRuO_4_ by this route, presumably due to the small oxygen partial pressure (∼2–3 ppm) in the argon gas. In an attempt to prepare a single-phase sample, the products from the reactions performed under flowing argon (which still contained some unreacted LiH) were sealed under vacuum in Pyrex ampules and heated at 375 °C for 24 h. PXRD data revealed that this led to the conversion of the LaSrCoRuO_5_/LaSrCoRuO_4_ mixture to a mixture of LaSrCoRuO_6–*x*_H_*y*_ oxyhydride phases similar to those in sample B. Thus, a sample of “LaSrCoRuO_4_” for detailed analysis, henceforth referred to as “sample C”, was prepared by heating ∼1.5 g of LaSrCoRuO_6_ with 8 mol equivalents of LiH under flowing argon at 375 °C for 1 period of 2 days, prior to washing to remove the lithium phases.

### Characterization of Sample A—LaSrCoRuO_3.2_H_1.9_

Thermogravimetric data collected while heating sample A under flowing oxygen (Figure S2) to oxidize it back to LaSrCoRuO_6_ (confirmed by PXRD) reveal a mass gain of 9.27%, consistent with an initial composition of LaSrCoRuO_3.2_. This is clearly an unrealistic composition, as it requires transition-metal oxidation states below +1, which we interpret as indicating the phase is an oxyhydride of the form LaSrCoRuO_3.2_H_*y*_. To determine the hydride content, iodometric titrations were performed to establish the transition-metal oxidation states, as described in detail in the Supporting Information, which indicated a composition of LaSrCoRuO_3.2_H_1.9_.

**Figure 1:**
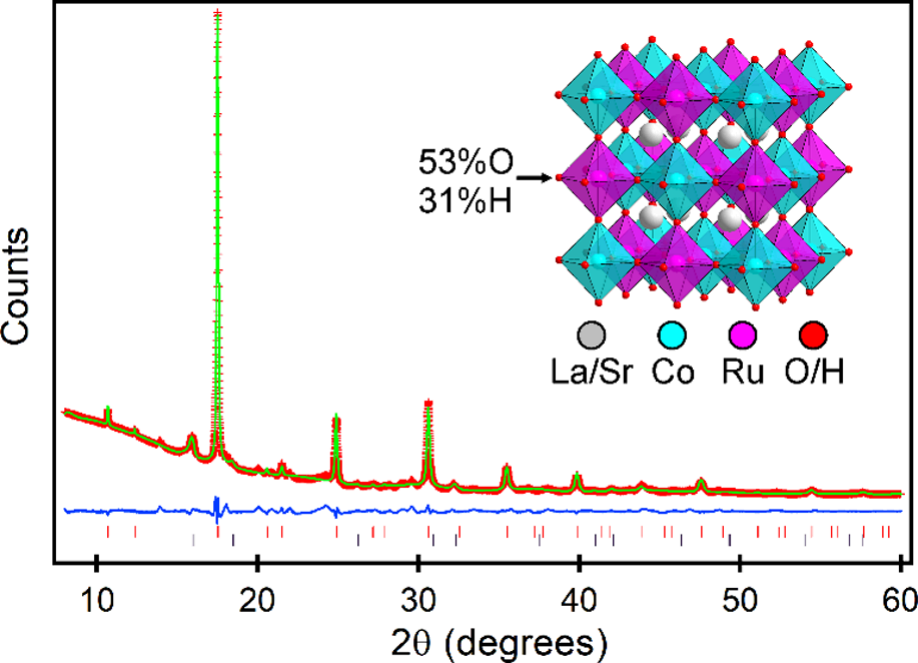
Observed calculated and difference plots from the structural refinement of LaSrCoRuO_3.2_H_1.9_ (sample A) against the SXRD data. Red tick marks indicate peak positions for the majority phase; black tick marks indicate a SrO secondary phase. The inset shows representation of the refined crystal structure.

SXRD data collected from LaSrCoRuO_3.2_H_1.9_ could be indexed using a face-centered unit cell, with reflection conditions consistent with space group *Fm*-3*m*. Thus, a structural model was constructed based on a cubic double perovskite with an anion site occupancy of O_0.53_H_0.32_ (as determined from the iodometric titrations), and this was refined against the SXRD data. Close inspection revealed additional diffraction peaks attributed to a secondary SrO phase, so this was added to the model. A good fit to the data was achieved, as shown in Figure 1 and described in detail in Table 1.

**Table 1:** Parameters from the Structural Refinement of LaSrCoRuO_3.2_H_1.9_ (Sample A) against SXRD Data Collected at Room Temperature

atom	x	y	z	fraction	*B*_iso_ (Å^2^)
La/Sr	1/4	1/4	1/4	0.5/0.5	3.05(4)
Co	0	0	0	1	2.82(5)
Ru	1/2	0	0	1	2.82(5)
O/H	1/2	0	1/4	0.53/0.32	3.18(3)
LaSrCoRuO_3.2_H_1.9_—space group *Fm*-3*m* (#225)					
*a* = 7.6679(1) Å, volume = 450.85(3) Å^3^					
formula weight = 439.64 g mol^–1^, *Z* = 4					
phase fraction = 96(1) mass %					
SrO - space group *Fm*-3*m* (#225)					
*a* = 5.1465(5) Å, volume = 136.31(4) Å^3^					
formula weight = 103.62 g mol^–1^, *Z* = 4					
phase fraction = 4(1) mass %					
radiation source: Synchrotron X-ray, λ = 0.8268 Å					
temperature: 298 K					
*R*_p_ = 2.30%, _w_R_p_ = 3.34%, *R*_Bragg_ = 1.03%					

### Characterization of Sample B—LaSrCoRuO_*x*_H_*y*_

Thermogravimetric data collected while heating sample B under flowing oxygen (Figure S3) to oxidize it back to LaSrCoRuO_6_ (confirmed by PXRD) reveals a mass gain of 5.86%, consistent with an initial composition of LaSrCoRuO_4.23_. However, mass spectrometry measurements monitoring the exhaust gas of the TGA instrument at *m*/*z* = 18 (Figure S4) indicated a release of water coincident with the oxidation of the sample, indicating that the sample contained an oxyhydride phase.

SXRD and NPD data collected from sample B could only be indexed as two phases: a body-centered tetragonal phase (*a* = 5.43 Å, *c* = 7.98 Å) and a face-centered cubic phase (*a* = 7.71 Å). A structural model was constructed consisting of a cubic LaSrCoRuO_6–*x*_ phase described in space group *Fm*-3*m* (#225) and a LaSrCoRuO_6–*x*_ phase described in space group *I*4/*m* (#87). This 2-phase model was refined against the SXRD and NPD data, with the anion occupancies in the two phases allowed to refine freely. On convergence, the two-phase model had an average composition of LaSrCoRuO_3.28_, significantly lower than the value determined from the TGA data (LaSrCoRuO_4.23_). To address this, hydride ions were added to the model, such that each anion site could be occupied by either oxide ions, hydride ions, or be vacant. This model was refined with the constraints that the total sample oxygen content agreed with the TGA data and that the sum of the oxide and hydride occupancies on any anion site could not be greater than unity.

**Figure 2:**
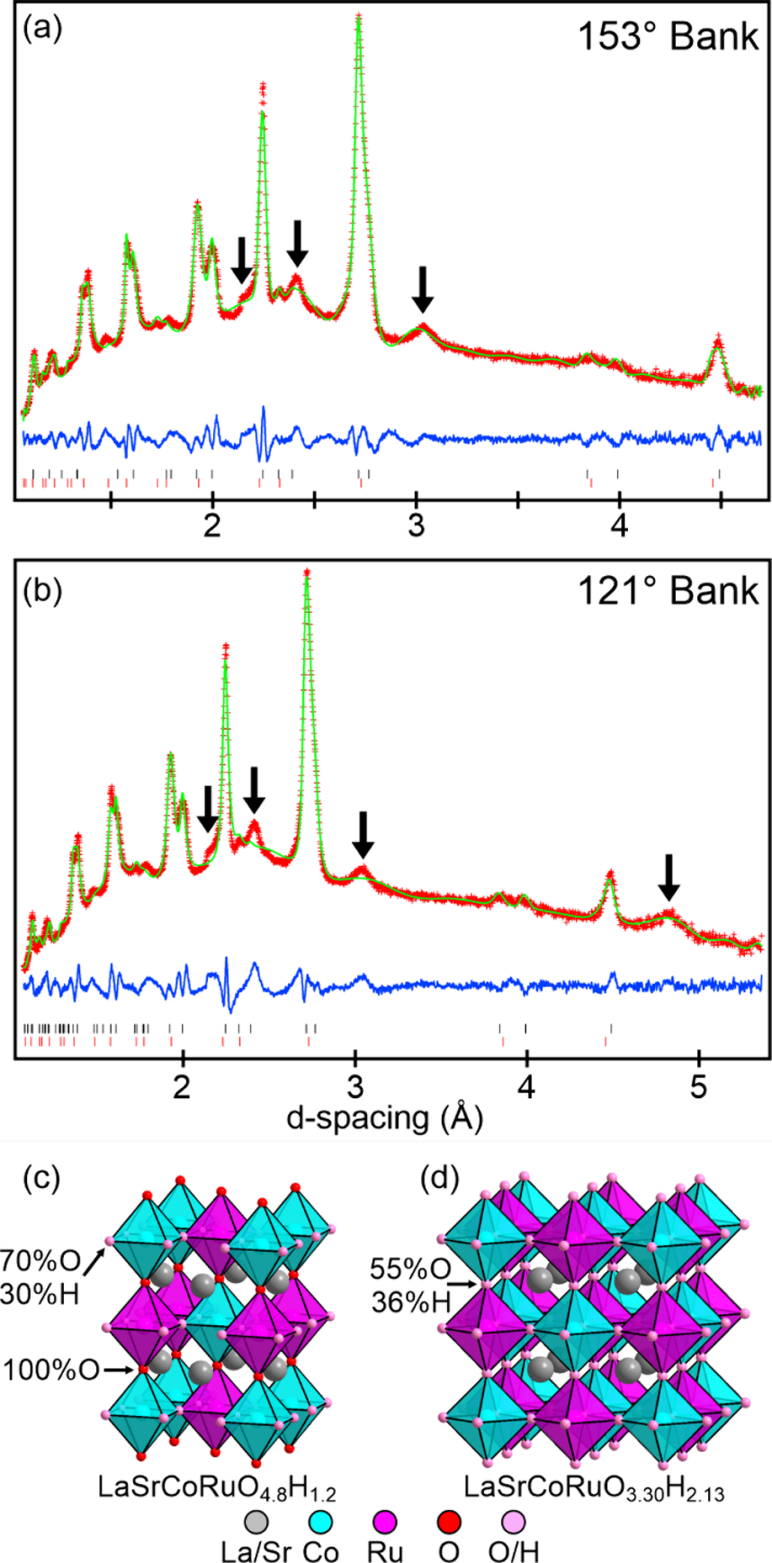
(a, b) Observed, calculated, and difference plots from the two-phase structural refinement of sample B against NPD data. Black tick marks indicate peak positions of LaSrCoRuO_4.8_H_1.2_, and red ticks indicate LaSrCoRuO_3.3_H_2.13_. Arrows indicate “supercell” reflections indicative of short-range anion order. Crystal structures of (c) LaSrCoRuO_4.8_H_1.2_ and (d) LaSrCoRuO_3.3_H_2.13._.

This model converged readily to yield compositions of LaSrCoRuO_4.80(1)_H_1.20(1)_ and LaSrCoRuO_3.30(6)_H_2.13(9)_ for the tetragonal and cubic phases, respectively. In addition, the tetragonal phase exhibits partial anion order with the 4*e* “axial” site fully occupied by oxygen, while the 8*h* “equatorial” site is a 0.7/0.3 mixture of oxygen and hydrogen as shown in Figure 2. This 2-phase model fits the SXRD data well as shown in Figure S6. However, close inspection of the NPD data (Figure 2) reveals a series of broad asymmetric peaks not indexed by the 2-phase model. Electron diffraction data (Figure S7) were collected to investigate the possibility that the broad features were due to short-range anion ordering. These data showed additional diffraction peaks indicating a doubling of the unit cell along the *x-* and *y*-axes of the tetragonal phase (ie. a 2√2 × 2√2 × 2 expansion relative to the primitive perovskite unit cell). Such a cell expansion allows the broad asymmetric features in the NPD data to be indexed. However, it was not possible to construct a structural model in the expanded unit cell that accounts for the additional diffraction features while simultaneously fitting the sharp features in the data. This suggests that the anion order responsible for the expanded cell is short ranged and incomplete. A detailed description of the structural parameters of tetragonal LaSrCoRuO_4.80(1)_H_1.20(1)_ and cubic LaSrCoRuO_3.30(6)_H_2.13(9)_ are given in Table 2.

**Table 2:** Parameters from the 2-phase structural refinement of Sample B against NPD data collected at room temperature

LaSrCoRuO_4.8_H_1.2_						
atom		*x*	*y*	*z*	fraction	*B*_iso_ (Å^2^)
La/Sr	4*d*	0	^1^/_2_	^1^/_4_	0.5/0.5	0.46(14)
Co	2*a*	0	0	0	1	0.97(14)
Ru	2*b*	^1^/_2_	^1^/_2_	0	1	0.97(14)
O/H(1)	8*h*	0.243(7)	0.257(8)	0	0.70(1)/0.30(1)	2.75(16)
O(2)	4e	0	0	^1^/_2_	1	2.75(16)
LaSrCoRuO_4.80(1)_H_1.20(2)_ – space group *I*4/*m* (#87)						
*a* = 5.4307(3) Å, *c* = 7.9842(8) Å, volume = 235.47(3) Å^3^						
formula weight = 464.54 g mol^–1^, *Z* = 2						
weight fraction= 65(1) mass %						

LaSrCoRuO_3.30_H_2.13_						
atom		*x*	*y*	*z*	fraction	*B*_iso_ (Å^2^)
La/Sr	8*c*	^1^/_4_	^1^/_4_	^1^/_4_	0.5/0.5	0.46(14)
Co	4*a*	0	0	0	1	0.97(14)
Ru	4*b*	^1^/_2_	0	0	1	0.97(14)
O/H	24e	^1^/_2_	0	^1^/_4_	0.55(1)/0.36(2)	2.75(16)
LaSrCoRuO_3.30(6)_H_2.13(9)_ – space group *Fm*-3*m* (#225)						
*a* = 7.7154(7) Å, volume = 459.28(7) Å^3^						
formula weight = 441.48 g mol–1, *Z* = 4						
weight fraction = 35(1) mass %						
radiation source: neutron time-of-flight						
temperature: 298 K						
*R*_p_ = 1.37 %, _w_*R*_p_ = 1.88%						

### Characterization of Sample C—LaSrCoRuO_4_

Thermogravimetric data collected while heating Sample C under flowing oxygen (Figure S9) to oxidize it back to LaSrCoRuO_6_ (confirmed by PXRD) reveals a mass gain of 6.39%, consistent with an initial composition of LaSrCoRuO_4.07_. NPD data collected from sample C could be indexed using a body-centered tetragonal unit cell (*a* = 5.67 Å, *c* = 6.88 Å) with a series of additional reflections attributable to the presence of small quantities of monoclinic LaSrCoRuO_5_.^15^ A 2-phase model was constructed combining a B-site ordered infinite layer phase described in space group *I*4/*mmm* and the reported structure of LaSrCoRuO_5_.

**Figure 3:**
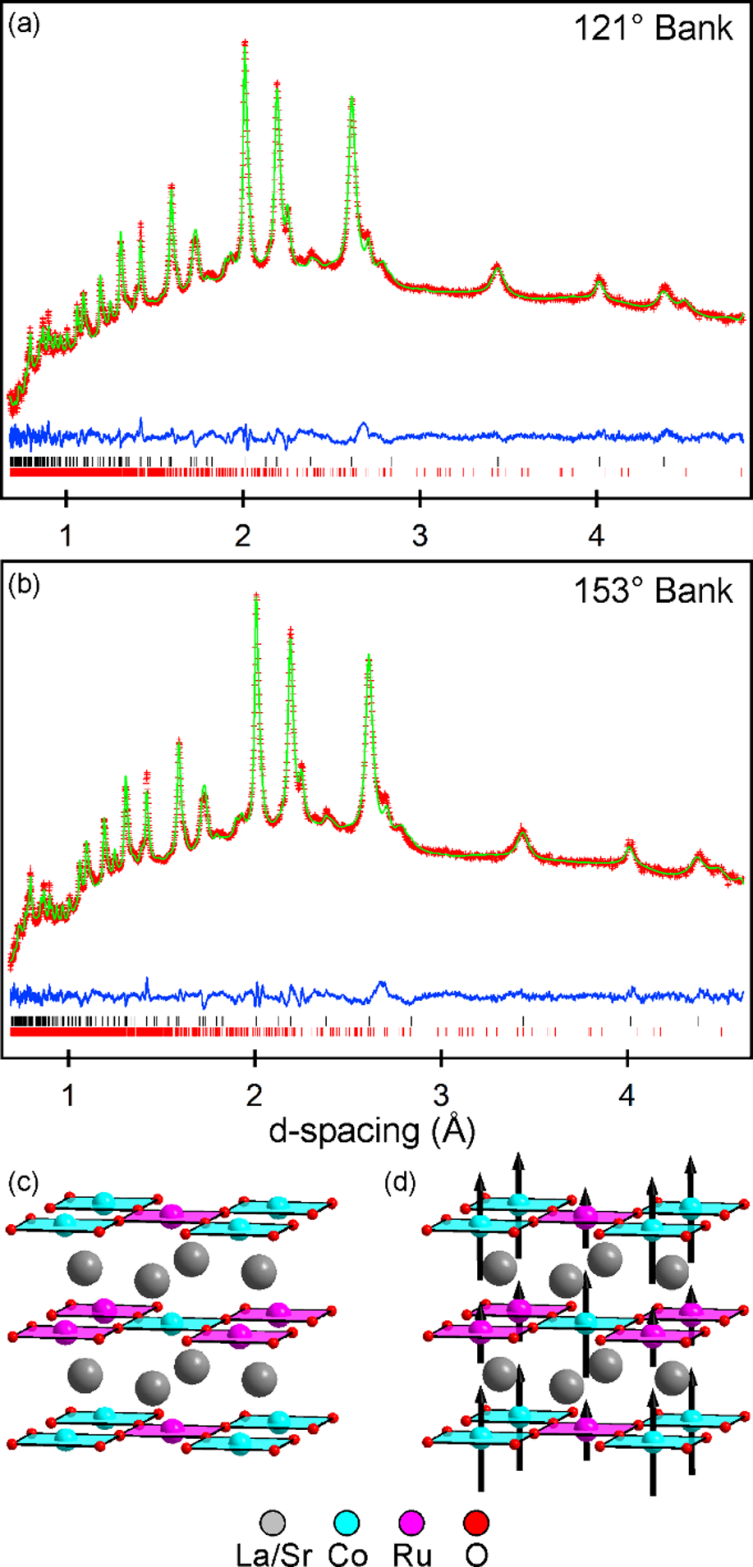
(a, b) Observed, calculated and difference plots from the 2-phase structural refinement of Sample C against NPD data collected at 300 K. Black ticks indicate peak positions of LaSrCoRuO_4_, red ticks LaSrCoRuO_5_. (c) Crystal structure of LaSrCoRuO_4_ at 300 K and (d) magnetic and crystal structure of LaSrCoRuO_4_ at 5 K, ordered moments: Co 1.62 μB; Ru 0.21 μB.

This model was refined against the NPD data to achieve a good fit. To investigate the possibility of cooperative tilting of the MO_4_ squares, a series of lower symmetry models were constructed. However, these lower symmetry models did not improve the fit to the data and were refined to give structures in which the Co–O–Ru bond angles remained 180°, within error, indicating the *I*4/*mmm* model, detailed in Table 3, is the best description of the structure. The fit to the NPD data is shown in Figure 3.

**Table 3:** Parameters from the Structural Refinement of LaSrCoRuO_4_ against NPD Data Collected at 300 K

atom	*x*	*y*	*z*	fraction	*B*_iso_
La/Sr	0	^1^/_2_	^1^/_4_	0.5/0.5	0.24(4)
Co	0	0	0	1	1.94(6)
Ru	0	0	^1^/_2_	1	1.94(6)
O	0.2519(4)	0.2519(4)	0	1	1.97(5)
LaSrCoRuO4 – space group *I*4/*mmm* (#139)					
*a* = 5.6786(2) Å, c = 6.8823(4) Å, volume = 221.93(2) Å^3^					
formula weight = 450.53 g mol^–1^, Z = 2					
weight fraction = 91.7(3) %					
LaSrCoRuO5 – space group *P*112_1_ (#4)					
*a* = 10.821(9) Å, *b* = 10.828(9) Å, *c* = 8.120(2) Å, γ = 91.02(3)°, volume = 951.2(1) Å^3^					
formula weight = 466.53 g mol^–1^, *Z* = 8					
weight fraction = 8.3(3) %					
radiation source: neutron time-of-flight					
temperature: 298 K					
_w_*R*_p_ = 2.01%; *R*p = 1.46%					

### Magnetic Characterization

Magnetization data collected from all reduced samples of LaSrCoRuO_6_ contained signals from small quantities of ferromagnetic impurities (most likely elemental cobalt), in common with many other topochemically reduced phases. Thus, a “ferromagnetic subtraction” method was used to measure the magnetization of the samples, described in detail in the Supporting Information.

**Figure 4:**
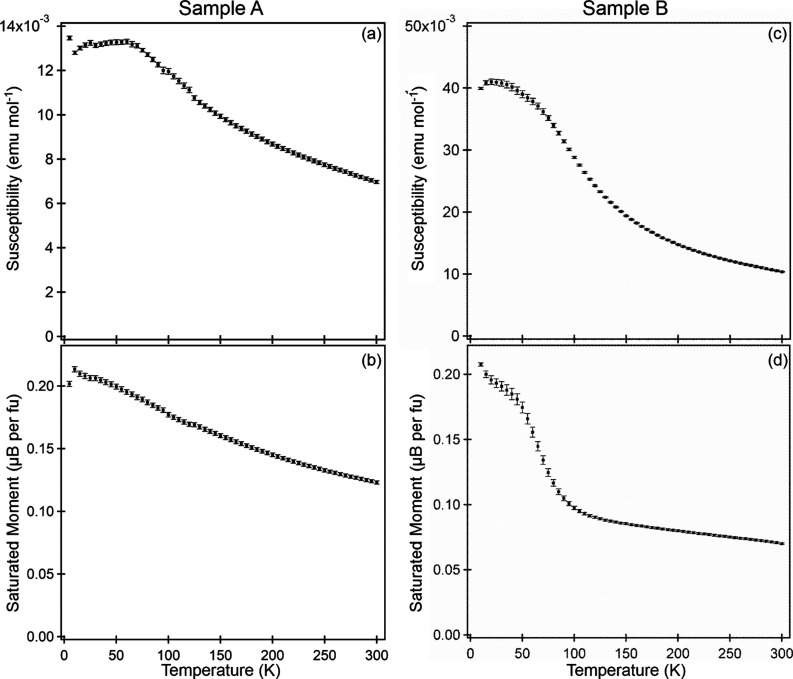
Magnetic susceptibility (a, c) and saturated ferromagnetic moment (b, d) data from sample A (LaSrCoRuO_3.2_H_1.9_) and sample B (LaSrCoRuO_4.8_H_1.2_ + LaSrCoRuO_3.30_H_2.13_), respectively.

Magnetic susceptibility data collected in this manner from sample A (Figure 4a) could be fitted by the Curie–Weiss law in the range 150 < *T*/K < 300 (Figure S11, Supporting Information). However, the parameters extracted, *C* = 3.58(2) cm^3^ K mol^–1^ and θ = −206(1) K, are much greater than those that can be accounted for by localized moments on the Co and Ru centers. The magnetization data exhibit a broad maximum at *T* ≈ 50 K, suggestive of the onset of antiferromagnetic order, but given the level of chemical disorder in the anion lattice of sample A (LaSrCoRuO_3.2_H_1.9_), it is likely that any magnetic order present is short ranged.

Equivalent magnetic susceptibility data collected from sample B (Figure 4c) can also be fit by the Curie–Weiss law in the range 100 < *T*/K < 300 (Figure S12, Supporting Information). Again, the extracted parameters, *C* = 2.48(3) cm^3^ K mol^–1^, θ = 8.5(9) K, cannot easily be accounted for by local moments on the transition-metal centers. Below *T* = 100 K, the saturated moment of the sample (Figure 4d) exhibits a sharp rise, while the susceptibility reaches a local maximum at *T* ≈ 15 K, suggesting the onset of canted antiferromagnetic order. However, NPD data collected from sample B at 5 K show no indication of long-range magnetic order, suggesting any magnetic order present is short ranged in nature.

**Figure 5:**
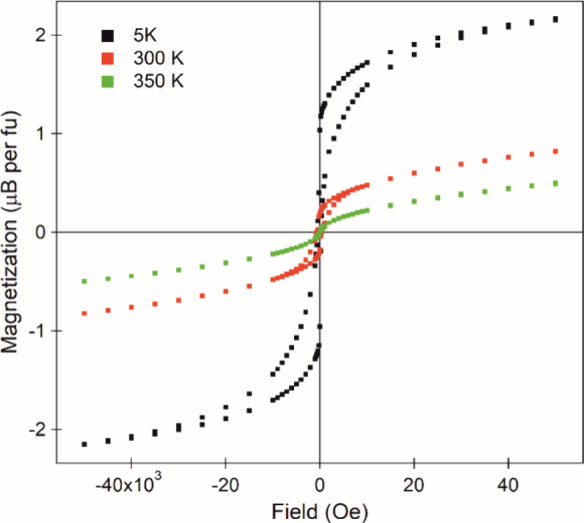
Magnetization-field data collected from sample C (LaSrCoRuO_4_) at 350, 300, and 5 K.

Magnetization data collected from sample C (LaSrCoRuO_4_) are qualitatively different to data from the other phases. Magnetization-field isotherms collected from sample C at 300 K (Figure 5) indicate that the sample is ferromagnetic with a saturated moment of 0.45 μB per fu. Analogous data collected at 350 K (the highest temperature attainable in our apparatus) are also indicative of ferromagnetic behavior (saturated moment 0.2 μB per fu). Magnetization data collected from sample C as a function of temperature in the range 5 < *T*/K < 350 using the “ferromagnetic subtraction” technique (Figure S13) exhibit no obvious transition, suggesting the onset of ferromagnetic behavior is above 350 K, although the likely presence of ferromagnetic elemental Co impurities make this value of *T*_c_ less certain.

As noted above, NPD data collected from sample C at 300 K could be fitted without the need to include a magnetic model. However, NPD data collected at 5 K show a strong enhancement of the [110] and [101] reflections. These enhanced intensities are best fit using a magnetic model described in space group 139.537 (*I*4/*mm*’*m*’) in which the moments on Co (1.62(6) μB) and Ru (0.21(7) μB) are aligned parallel to the *z*-axis, to yield a total ordered moment of 1.83(9) μB, in line with the saturated moment of 1.75 μB per fu extracted from the magnetization data. Full details of this structural refinement are given in the Supporting Information. Refinement of this magnetic model against the NPD data collected at 300 K yields ordered moments on Co and Ru that are smaller than the associated errors, indicating that while magnetization data indicate that LaSrCoRuO_4_ is ferromagnetically ordered at 300 K, the magnetic scattering associated with this phase is not observable in the absence of an applied magnetic field.

## Discussion

### Reactivity

**Scheme 1:**
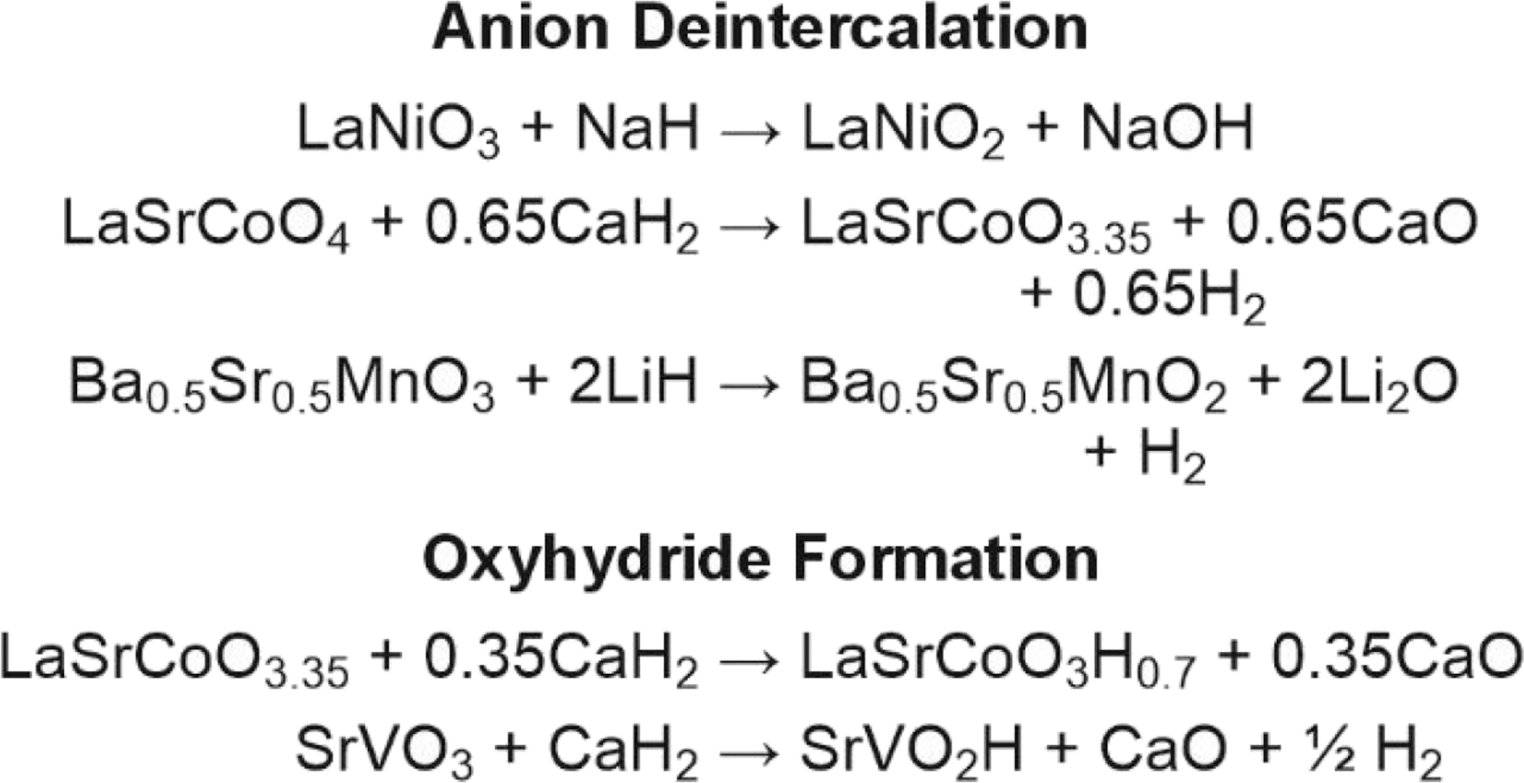
Reactions of Binary Hydrides with Complex Oxides

The reactions that occur between binary metal hydrides (NaH, LiH, CaH_2_) and complex transition-metal oxides yield products that can be organized into two main classes: (i) anion deficient oxide phases (ii) oxyhydride phases, which contain both O^2–^ and H^–^ anions. Literature examples of the reactions that form these two product types are given in Scheme 1.^4,10,16,17^

The reactions in Scheme 1 show that the hydride ions in NaH act as 2-electron reducing agents in anion deintercalation reactions (NaH → NaOH),^4^ while the hydride ions in LiH and CaH_2_ act as 1-electron reducing agents (2LiH → Li_2_O + H_2_; CaH_2_ → CaO + H_2_)^10,16^ with hydrogen gas produced during reactions which yield anion deficient oxide phases.

Scheme 1 also shows that oxyhydride formation reactions are observed to occur either (a) via a simple redox-neutral anion exchange process as seen for LaSrCoO_3.35_ → LaSrCoO_3_H_0.7_ with no generation of hydrogen,^10,18,19^ or (b) as a combined reduction + anion-exchange reaction as observed for SrVO_3_ → SrVO_2_H, with the generation of H_2_ gas.^17^

The redox-neutral anion-exchange process that forms oxyhydride compounds by route (a) starts with an anion-deficient oxide phase, which is usually produced from an oxygen-stoichiometric compound by the action of the same binary hydride reagent that affects the hydride-for-oxide anion exchange (e.g., CaH_2_). The result is a reaction sequence such as LaSrCoO_4_ → LaSrCoO_3.35_ → LaSrCoO_3_H_0.7_, which only differs in total from the combined reduce + anion-exchange reactions in route (b) by the observation of an isolatable, anion-deficient all-oxide intermediate phase. Thus, the two observed topochemical routes for oxyhydride formation, (a) reduce-then-exchange or (b) reduce-and-exchange, can be considered as the extremes on a “spectrum” of oxyhydride formation reactions which differ only by how separate the “reduce” and “exchange” processes are.

**Figure 6:**
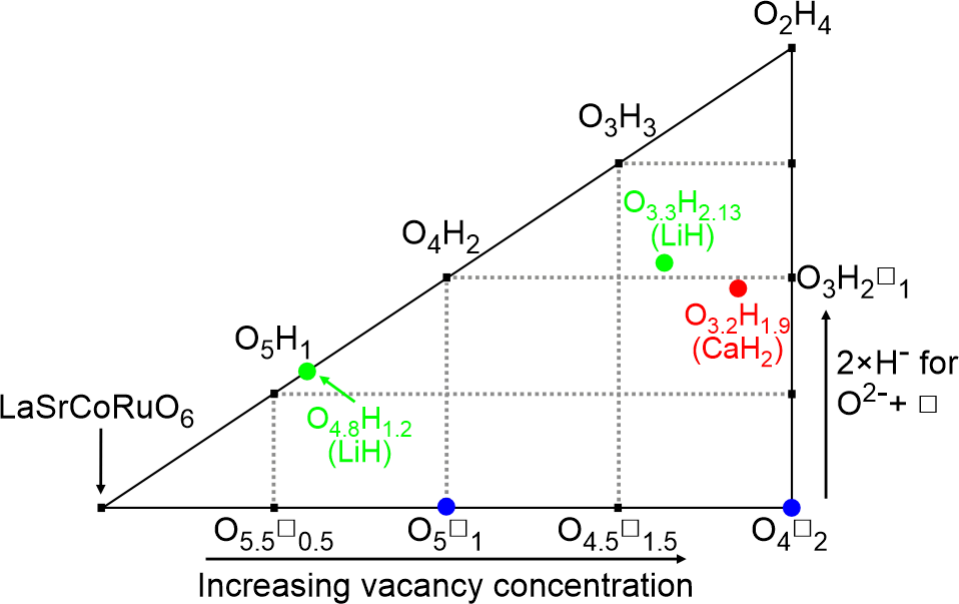
Phase diagram showing the topochemically reduced phases derived from LaSrCoRuO_6_ via anion deintercalation and hydride-for-oxide anion exchange.

In the context of the discussion above, the reactions between LaSrCoRuO_6_ and the binary hydrides LiH and CaH_2_ provide an interesting platform to study this type of chemistry. Figure 6 shows a phase diagram for the products of these topochemical reactions. Starting at stoichiometric LaSrCoRuO_6_ on the left-hand side, moving horizontally across the diagram to the right corresponds to the reductive deintercalation of oxide ions to form LaSrCoRuO_6–*x*_ phases. Thus, the two experimentally observed anion vacancy-ordered phases LaSrCoRuO_5_ and LaSrCoRuO_4_ are located on the bottom edge of the diagram (blue markers). Moving vertically up the diagram corresponds to the redox-neutral anion exchange of one O^2–^ oxide ion and one vacancy for two H^–^ hydride ions. Thus, the oxyhydride phases present in samples A (red marker) and B (green markers) lie away from the bottom axis. The phase diagram is bounded by a “top” edge, which corresponds to completely filling the anion sites with a combination of oxide and/or hydride ions, i.e., LaSrCoRuO_*x*_H_*y*_ phases where *x* + *y* = 6. It therefore follows that phases not located on the top edge of the diagram contain anion vacancies. It should also be noted that the diagram is arranged so that the oxidation states of the transition metals decline on moving to the right across the diagram.

The distribution of the five LaSrCoRuO_6–*x*_H_*y*_ phases experimentally isolated in this work show that unless efforts are made to actively lower the partial pressure of hydrogen (as was the case for sample C) reactions between LaSrCoRuO_6_ and LiH or CaH_2_ yield oxyhydride phases directly, with no evidence for the presence of anion-deficient all-oxide intermediates. This contrasts with the behavior of several previously studied cobalt oxide systems. For example, as noted above, LaSrCoO_4_ reacts with CaH_2_ at *T* ≈ 340 °C to form LaSrCoO_3.35_, which is then converted to LaSrCoO_3_H_0.7_ by further reaction with CaH_2_ at *T* > 450 °C via the (a) reduce-then-exchange route.^10,18,19^ Likewise, the Co/Ru phase LaSr_3_CoRuO_8_ is converted to LaSr_3_CoRuO_6_ on reaction with CaH_2_ at 425 °C before further reaction at 450 °C yields LaSr_3_CoRuO_4_H_4_.^20^

**Figure 7:**
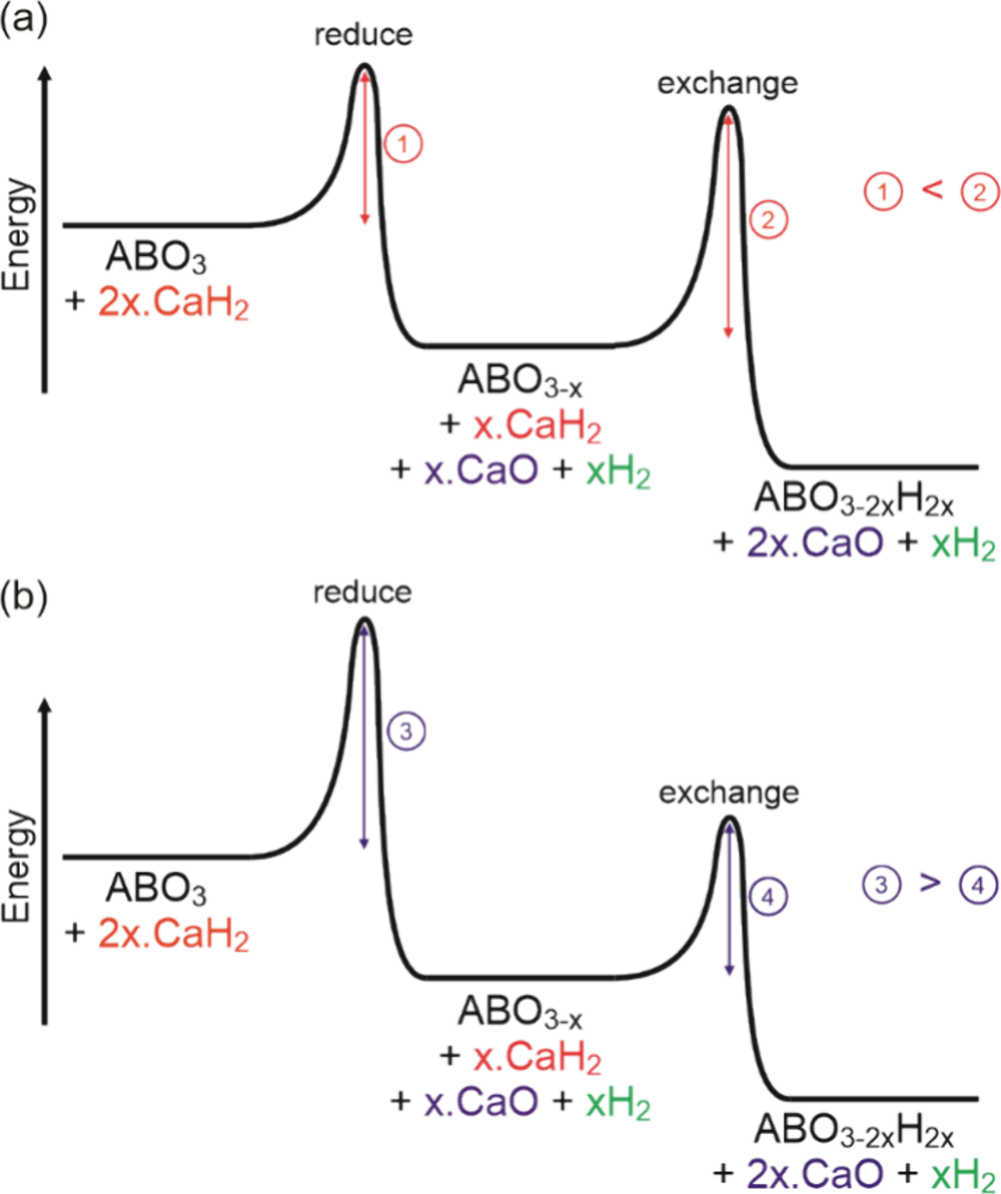
Schematic diagram showing the relative activation energies in the two classes of topochemical oxyhydride formation: (a) reduce-then-exchange and (b) reduce-and-exchange.

In the two examples described above, the reductive anion deintercalation step occurs at a lower temperature than the anion-exchange step. This indicates that the activation energy for the reductive step is lower than that of the anion-exchange step, as shown in the schematic energy diagram in Figure 7a. Thus, by supplying sufficient energy to overcome the first barrier but not the second, an anion-deficient all-oxide phase can be isolated.

The direct conversion of LaSrCoRuO_6_ to LaSrCoRuO_6-x_H_*y*_ oxyhydride phases, without any sign of anion-deficient all-oxide intermediates, indicates that, in this system, the activation energy for the anion-exchange step is equal to, or less than, the activation energy for the reductive deintercalation step, as shown in Figure 7b. As a result, as soon as sufficient energy is supplied to overcome the first barrier to form an anion deficient phase, there is enough energy to overcome the second barrier to convert it to an oxyhydride phase, so that no anion-deficient intermediate phase accumulates. This scenario of sequential reactions is supported by the observation that LaSrCoRuO_6–*x*_ phases can be isolated from reactions between LaSrCoRuO_6_ and LiH if the second reaction step (anion-exchange) is prevented, by keeping the P(H_2_) in the system low (i.e., performing the reaction under flowing argon), but that such “intermediates” will react rapidly with LiH to yield LaSrCoRuO_6–*x*_H_*y*_ phases when heated in a sealed system and the P(H_2_) is allowed to rise.

The origin of the contrasting reaction pathways adopted by LaSrCoRuO_6_ (reduce-and-exchange) and the analogous *n* = 1 Ruddlesden–Popper phase, LaSr_3_CoRuO_8_ (reduce-then-exchange)^20^ is unclear. However, it should be noted that the reactivity of LaSrCoRuO_6_ was enhanced by rapid quenching, in a manner analogous to that reported for LaSrNiRuO_6_.^21^ This quenching process could have the effect of lowering the activation barriers for both reductive anion deintercalation and anion exchange, making them more similar and resulting in the observed reactivity.

As noted above, it is clear that the hydrogen partial pressure influences the progress of hydride-for-oxide anion-exchange in the LaSrCoRuO_6–*x*_H_*y*_ system, and similar effects have been seen during the preparation of other oxyhydride phases. For example, the hydrogen content in LnSrCoO_3+*x*_H_*y*_ phases has been observed to be strongly dependent on the hydrogen pressure in the system during synthesis.^22^ Similarly, the hydrogen content in BaTiO_3–*x*_H_*y*_ phases is also observed to be strongly dependent on the synthesis procedure used.^23−25^ However, despite the obvious influence of P(H_2_) on the formation of oxyhydride phases, the microscopic role of H_2_ gas in the formation of oxyhydride phases is not immediately clear.

While it is tempting to assume the role of the hydrogen atmosphere is to supply the hydrogen which is simply inserted into vacant oxide phases (i.e., ABO_3–*x*_ + H_2_ → ABO_3–*x*_H_*y*_), there is limited evidence for this occurring. Indeed, in many cases direct reaction of hydrogen gas with complex transition-metal oxides yields anion-deficient phases, which can only be converted to oxyhydride phases on the addition of binary metal hydrides, not further reaction with hydrogen gas alone.^18,19^ Furthermore, the simple insertion of hydrogen requires the formal oxidation of the vacant oxide phase with hydrogen, which is both chemically unlikely and inconsistent with the observation of redox-neutral anion exchange processes. However, while there is little evidence for direct oxidative insertion of hydrogen, there is evidence for some interaction between hydrogen gas and oxyhydride phases, most notably in the observation of H/D exchange when D_2_ is passed over BaTiO_3–*x*_H_*y*_ at 400 °C.^23^

**Scheme 2:**
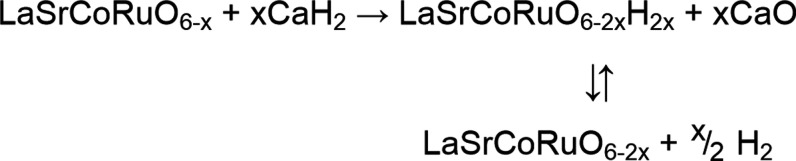
Role of H_2_ gas in Stabilizing Oxyhydride Phases during Synthesis

Given the arguments and observations above, we propose that the role of the hydrogen atmosphere in the synthesis of transition-metal oxyhydrides is not to supply hydrogen for the anion exchange step but to stabilize the oxyhydride phases formed against hydrogen loss. Specifically, we propose that in the LaSrCoRu(O/H)_*n*_ system (and the majority of other topochemically prepared transition-metal oxyhydrides), the hydride-for-oxide anion exchange reaction, which converts LaSrCoRuO_6–*x*_ to LaSrCoRuO_6–2*x*_H_2*x*_, occurs via an all-solid-state route in which oxide and hydride ions are exchanged between LaSrCoRuO_6–*x*_ and LiH or CaH_2_ without the formation of hydrogen gas. We further propose that at the reaction temperature, the oxyhydrides formed are likely to be unstable with respect to entropy-driven H_2_ loss as shown in reaction Scheme 2. We propose that the role of the H_2_ gas is to drive the resulting equilibrium towards hydrogen-rich oxyhydride phases, preventing their decomposition back to all-oxide materials. Such a scenario is consistent with the observation that LaSrCoRuO_*x*_H_*y*_ phases do not form under low P(H_2_) but cannot be made by the direct action of H_2_ on LaSrCoRuO_6_ in the absence of LiH or CaH_2_. Further support for this scenario comes from the observed release of H_2_ from sample B when heated under N_2_ at 375 °C, as shown in Figure S5. While we have described the role of H_2_ gas specifically in the synthesis of LaSrCoRuO_*x*_H_*y*_ phases, we think it is likely to apply widely in the topochemical synthesis of transition-metal oxyhydrides.

### Magnetism

**Figure 8:**
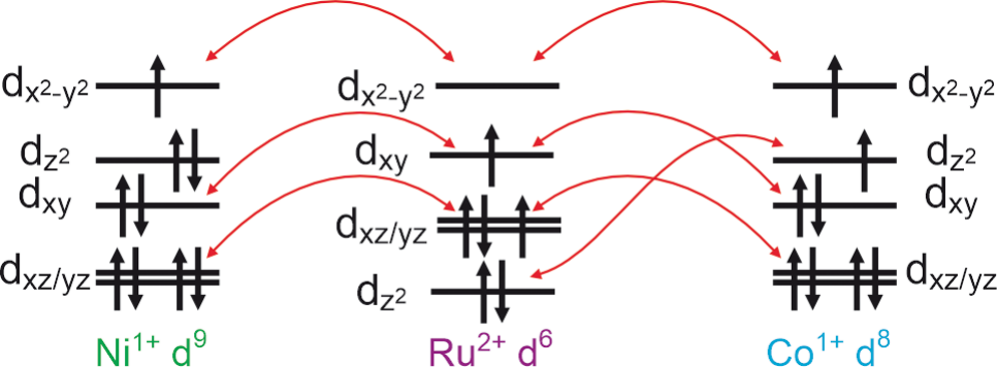
Superexchange interactions between square-planar Ni^1+^ or Co^1+^ and Ru^2+^. Red arrows indicate ferromagnetic interactions.

The ferromagnetic behavior of LaSrCoRuO_4_ is similar to that of the analogous nickel phase, LaSrNiRuO_4_ (*T*_c_ = 250 K)^26^ and the related reduced Ruddlesden–Popper oxide phases LaSr_3_NiRuO_6_ (*T*_c_ = 105 K) and LaSr_2_NiRuO_5_ (*T*_c_ = 200 K).^27^ The ferromagnetic behavior of LaSrNiRuO_6_ can be rationalized on the basis of superexchange couplings between S = 1/2 Ni^1+^ and S = 1 Ru^2+^ as shown in Figure 8.

An analogous case can be made for the ferromagnetic behavior of LaSrCoRuO_4_. However, in this case, the d^8^ electron count of Co^1+^ leads to an S = 1 configuration, which results in four ferromagnetic orbital couplings (Figure 8), compared to three in LaSrNiRuO_4_, consistent with the higher Curie temperature for the cobalt phase (*T*_c_ > 350 K) compared to the nickel phase (*T*_c_ = 250 K).^26^

The magnetization data collected from the LaSrCoRuO_*x*_H_*y*_ phases in sample A and sample B are consistent with antiferromagnetic interactions between localized moments on Co and Ru. This behavior is hard to explain on the basis of simple superexchange interactions because when 5 or 6 coordinate, the Co^1+/2+^ centers would be expected to adopt (t_2g_)^5/6^(e_g_)^2^ configurations, which should couple to the Ru^2+/3+^ (t_2g_)^5/6^(e_g_)^0^ cations via strong ferromagnetic σ–superexchange interactions. Similar unexpected antiferromagnetic couplings are also observed between the (t_2g_)^5^(e_g_)^2^ Co^2+^ and (t_2g_)^3^(e_g_)^0^ Ru^5+^ centers in the parent LaSrCoRuO_6_ oxide phase,^13^ adding to the body of evidence that many 3*d*/4*d* transition-metal oxide system exhibit magnetic behavior that cannot be rationalized by simple superexchange couplings.

## Conclusions

Reaction between LaSrCoRuO_6_ and LiH or CaH_2_ yields LaSrCoRuO_*x*_H_*y*_ oxyhydride phases if the P(H_2_) in the system is sufficient to stabilize these products with respect to hydrogen loss. Conversely, reaction between LaSrCoRuO_6_ and LiH under low P(H_2_) conditions yields the infinite layer phase LaSrCoRuO_4_, which exhibits insulating ferromagnetic behavior due to strong σ-type superexchange between Co^1+^ and Ru^2+^ centers. The influence of the local P(H_2_) on the competition between the formation of vacant oxides and oxyhydrides offers a method to control topochemical reductions to produce products with the desired oxide/hydride anion ratios.

## Supporting Information Available

Complete structural characterization of LaSrCoRuO_6_; thermogravimetric data from samples A, B and C; electron diffraction data from sample B; description of iodometric titration of sample A; description of the ferromagnetic subtraction method; complete magnetic characterization of sample C (PDF)
