# Supplementary material for: Competition between Anion-Deficient Oxide and Oxyhydride Phases during the Topochemical Reduction of LaSrCoRuO6
Source: Inorg Chem. 2024 Jun 28;63(28):12910–9. doi: 10.1021/acs.inorgchem.4c01568 (PMC11256754; doi:10.1021/acs.inorgchem.4c01568)
Supplement: Supplementary file 1 — ic4c01568_si_001.pdf [file ic4c01568_si_001.pdf]

Competition between anion-deficient oxide and oxyhydride phases during the topochemical reduction of $LaSrCoRuO_6$.

Zhilin Liang,[†] Maria Batuk,[Ψ] Fabio Orlandi,[§] Pascal Manuel,[§] Joke Hadermann[Ψ] and Michael A. Hayward[†*].

[†] Department of Chemistry, University of Oxford, Inorganic Chemistry Laboratory, South Parks Road, Oxford, OX1 3QR, UK.

[Ψ] EMAT, University of Antwerp, Groenenborgerlaan 171, B-2020 Antwerp, Belgium.

[§] ISIS Facility, Rutherford Appleton Laboratory, Chilton, Oxon OX11 0QX, UK.

* michael.hayward@chem.ox.c.uk

Supporting Information

Table of Contents

1. Structural Characterisation of $LaSrCoRuO_6$.
Figure S1. Observed, calculated and difference plots from the structural refinement of a $P2_1/n$ symmetry model against SXRD data collected from $LaSrCoRuO_6$ at room temperature.
Table S1. Parameters extracted from the structural refinement of $LaSrCoRuO_6$ against SXRD data.

2. Thermogravimetric analysis of Sample A.
Figure S2. Thermogravimetric data collected while heating Sample A under flowing oxygen.

3. Iodometric Titration of Sample A

4. Thermogravimetric analysis of Sample B.
Figure S3. Thermogravimetric data collected while heating Sample B under flowing oxygen.
Figure S4. Thermogravimetric data (top) and m/z = 18 mass-spectrum signal (bottom) collected as a function of temperature during the reoxidation of sample B back to $LaSrCoRuO_6$ under oxygen.
Figure S5. Thermogravimetric data (top) and m/z = 18, m/z = 2 mass-spectrum signals (bottom) collected as a function of temperature during the reoxidation of sample B back to $LaSrCoRuO_6$ under N_2.

5. Structural characterization of Sample B.
Figure S6. Observed, calculated and difference plots from the structural refinement of Sample B against SXRD data using model described in Table 2 in the main text.
Figure S7. Electron diffraction data collected from Sample B.

6. Synthesis of Sample C.
Figure S8. Schematic diagram of the experimental setup for the synthesis of Sample C.

7. Thermogravimetric analysis of Sample C.
Figure S9. Thermogravimetric data collected while heating Sample C under flowing oxygen.

8. Magnetic measurements in the presence of elemental Co impurities via the 'ferrosubtraction' method.
Figure S10. Magnetisation of Sample A measured as a function of applied field at 300 K.

9. Magnetic Characterization of Sample A.
Figure S11. Plot of inverse magnetic susceptibility against temperature for Sample A. Linear fit for T > 150 K, consistent with Curie-Weiss law.

10. Magnetic Characterization of Sample B.
Figure S12. Plot of inverse magnetic susceptibility against temperature for Sample B. Linear fit for T > 150 K, consistent with Curie-Weiss law.

11. Magnetic Characterization of Sample C.
Figure S13. Magnetization data collected from Sample C using the ferrosubtraction procedure.
Figure S14. Observed calculated and difference plots from the structural and magnetic refinement of $LaSrCoRuO_4$ against NPD data collected at 5 K.
Table S2. Parameters from the structural and magnetic refinement of $LaSrCoRuO_4$ against NPD data collected at 5 K.

1. Structural Characterisation of $LaSrCoRuO_6$.

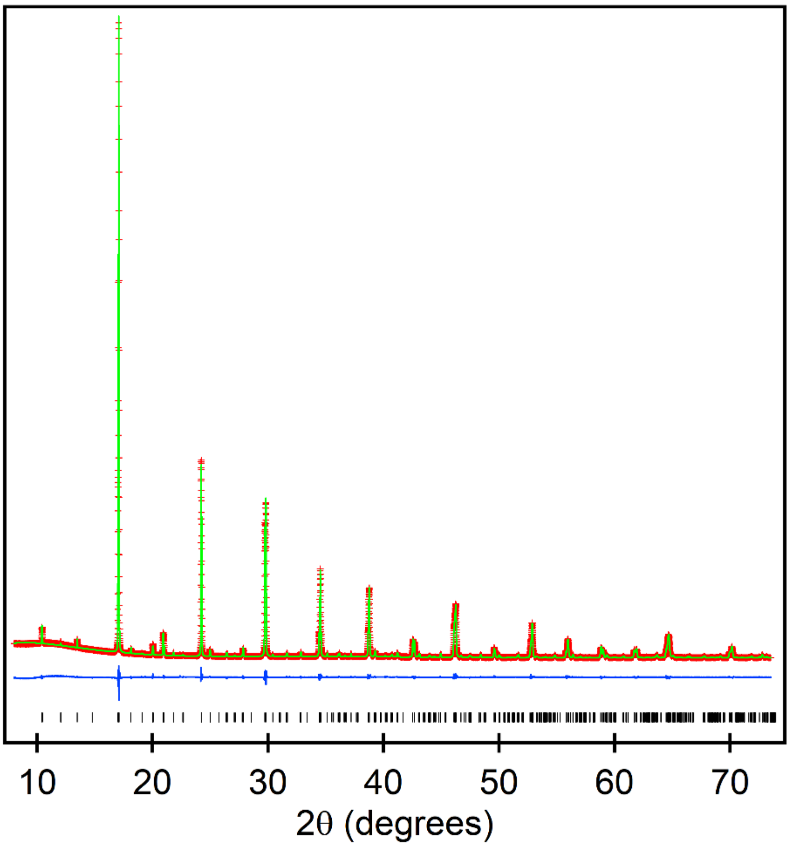

Figure S1. Observed, calculated and difference plots from the structural refinement of a $P2_1/n$ symmetry model against SXRD data collected from $LaSrCoRuO_6$ at room temperature.

$LaSrCoRuO_6$					
Atom	*x*	*y*	*z*	Fraction	B_{iso}
La/Sr	0.0054(1)	0.0224(1)	0.2506(1)	0.5/0.5	0.074(5)
Ni/Ru(1)	½	0	½	0.987(3)/0.013(3)	0.035(2)
Ru/Ni(2)	½	0	0	0.987(3)/0.013(3)	0.035(2)
O(1)	0.287(1)	0.274(1)	0.032(1)	1	0.086(3)
O(2)	0.233(1)	0.778(1)	0.027(1)	1	0.086(3)
O(3)	0.932(1)	0.494(1)	0.251(1)	1	0.086(3)
Space Group $P2_1/n$ Lattice Parameters a = 5.5906(1) Å, b = 5.5663(1) Å, c = 7.8791(1) Å, β = 89.992(1), volume = 245.20(1) Å^3					
Radiation source: Synchrotron X-ray, λ = 0.8268 Å Temperature: 298 K Rp = 3.29%, Rwp = 4.26%, R_{Bragg} = 1.34%					

Table S1. Parameters extracted from the structural refinement of $LaSrCoRuO_6$ against SXRD data.

2. Thermogravimetric analysis of Sample A.

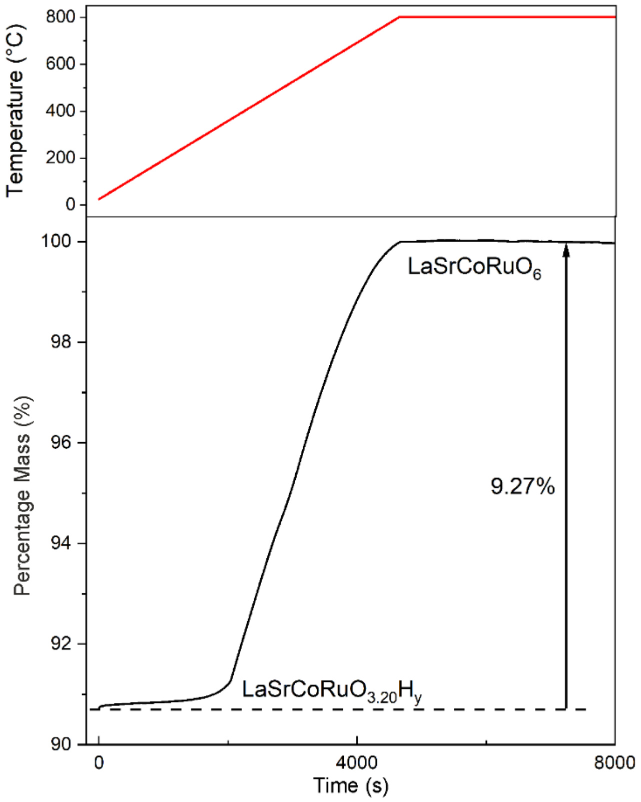

Figure S2. Thermogravimetric data collected while heating Sample A under flowing oxygen.

3. Iodometric Titration of Sample A

The hydride content of Sample A was determined via Iodometric titration. As the oxidation states of cobalt and ruthenium are equal or less than 2, the major redox reactions of the oxidative iodometric titration are as follows:

$$Co^{a+} + {}^{(2-a)}/_{2}\, I_2 \rightarrow Co^{2+} + (2-a)\, I^-$$

$$Ru^{b+} + {}^{(2-b)}/_{2}\, I_2 \rightarrow Ru^{2+} + (2-b)\, I^-$$

10 ml of a standardised 0.00166 M KIO_3 solution was pipetted into a 3-necked flask under flowing argon and an excess of KI (~70 mg) was added, to liberate 4.98×10^{-5} moles of I_2. To this solution 10 ml of aqueous, 2 M HCl was added along with a carefully weighed portion of Sample A (~30 mg). The solution was stirred under argon and Sample A dissolved in the solution, consuming I_2 according to the reaction scheme above. The quantity of unreacted I_2 was then determined by titration with a standardised solution of $Na_2S_2O_4$, using starch as an indicator. A constant argon flow was maintained to avoid oxidation of the samples by air throughout the whole titration process. The titration was repeated 5 times to establish the quantity of I_2 consumed on dissolution of Sample A, and thus the average oxidation states of the transition metals. Combining these values and the oxygen content obtained from the TGA experiment, the hydride content of Sample A can be determined.

4. Thermogravimetric analysis of Sample B.

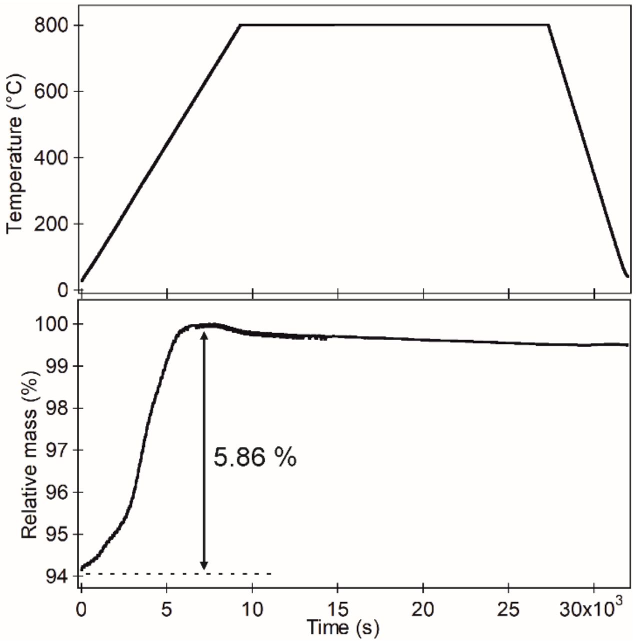

Figure S3. Thermogravimetric data collected while heating Sample B under flowing oxygen.

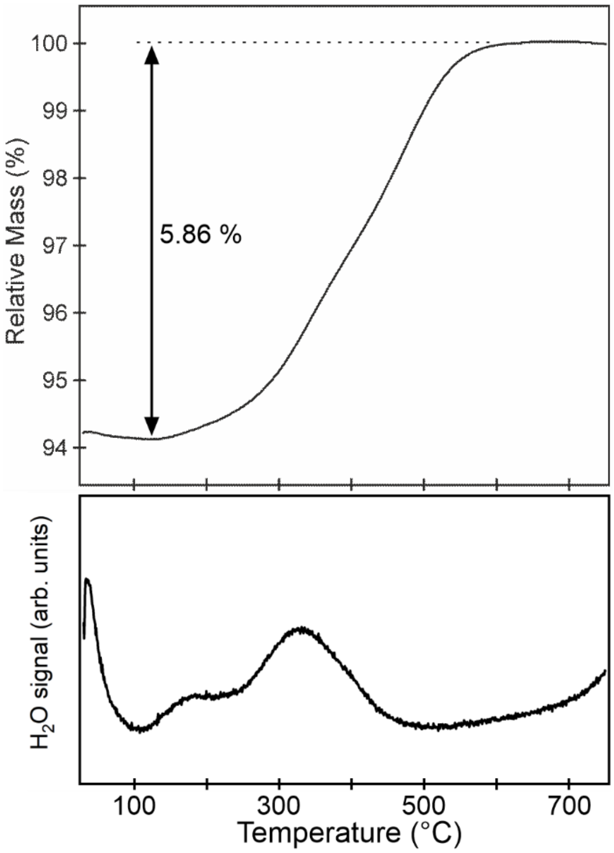

Figure S4. Thermogravimetric data (top) and m/z = 18 mass-spectrum signal (bottom) collected as a function of temperature during the reoxidation of sample B back to $LaSrCoRuO_6$ under oxygen.

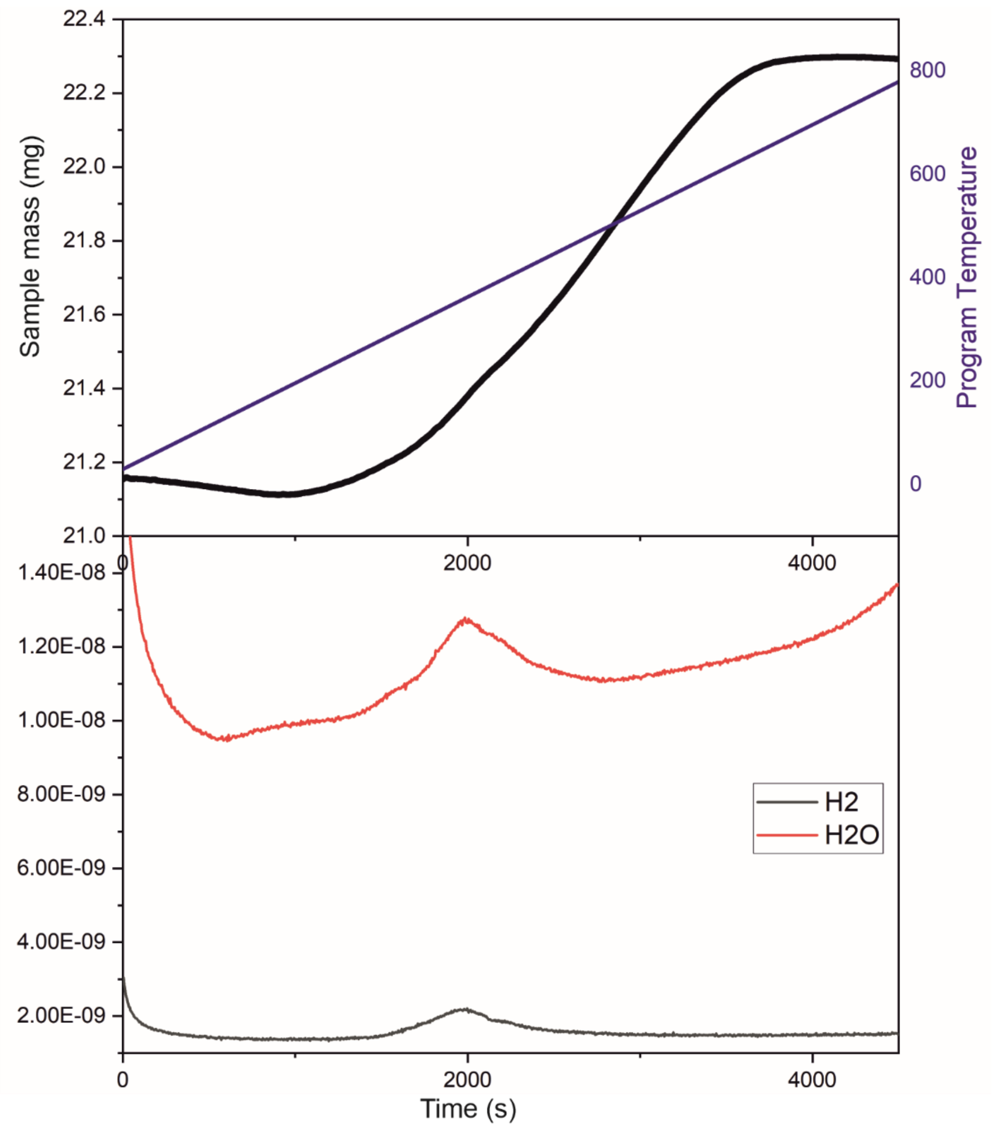

Figure S5. Thermogravimetric data (top) and m/z = 18, m/z = 2 mass-spectrum signals (bottom) collected as a function of temperature during the reoxidation of sample B back to $LaSrCoRuO_6$ under N_2 gas.

5. Structural characterization of Sample B.

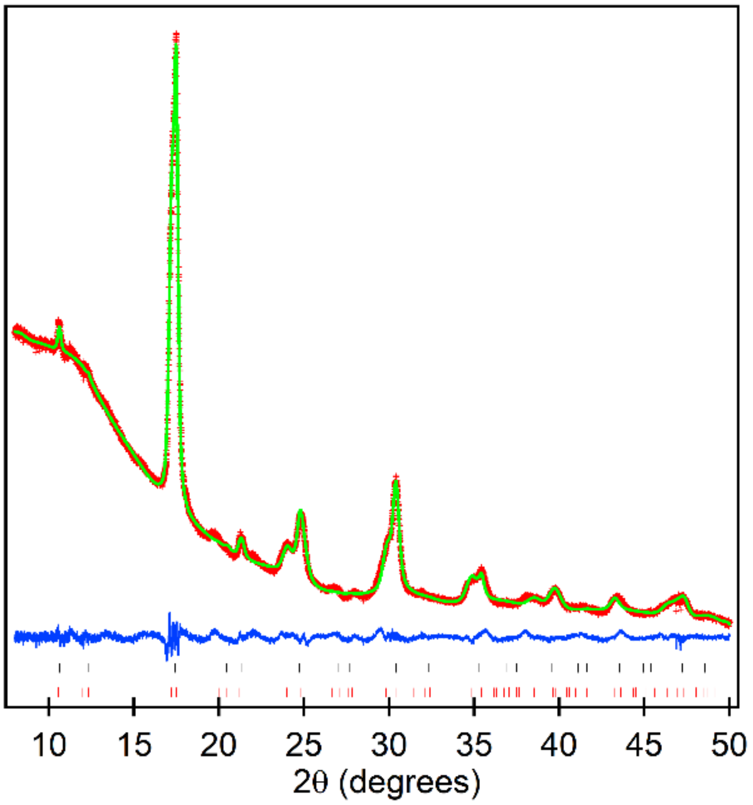

Figure S6. Observed, calculated and difference plots from the structural refinement of Sample B against SXRD data using model described in Table 2 in the main text. Red tick marks indicate peak positions of $LaSrCoRuO_{4.8}H_{1.2}$, black ticks $LaSrCoRuO_{3.3}H_{2.13}$.

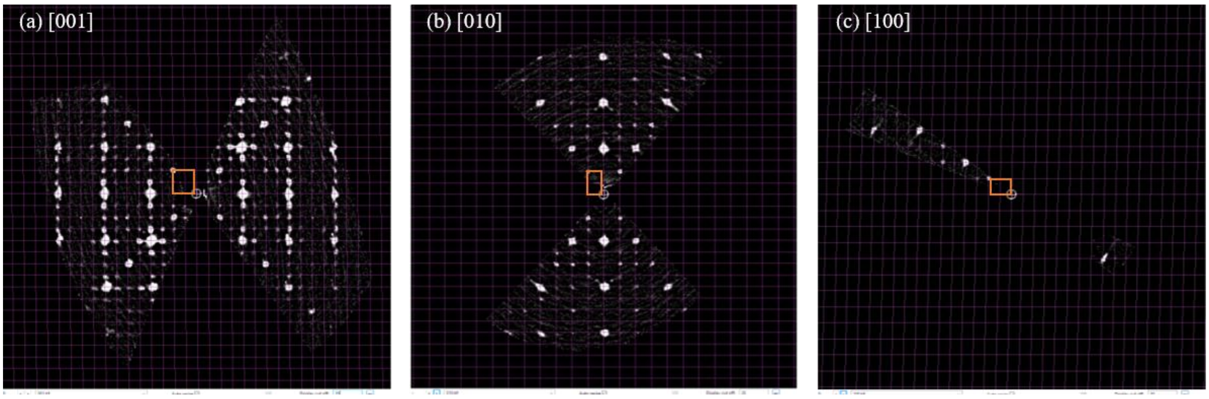

Figure S7. Electron diffraction data collected from Sample B. Additional reflections indicate a 2 × 2 × 1 geometric expansion of the unit cell of tetragonal $LaSrCoRuO_{4.8}H_{1.2}$.

6. Synthesis of Sample C.

Sample C was prepared by grinding $LaSrCoRuO_6$ with 8 mole equivalents of LiH in an argon filled glove box. The resulting mixture was then poured into an open-ended Pyrex tube that was placed within a silica flow-tube which could be sealed at each end with valves, so that the flow-tube assembly could be inserted into a clam-shell furnace while maintaining an argon atmosphere over the sample mixture. The flow-tube was then purged with argon for 20 minutes before being heated as described below, under a constant flow of argon, as shown schematically in Figure S8.

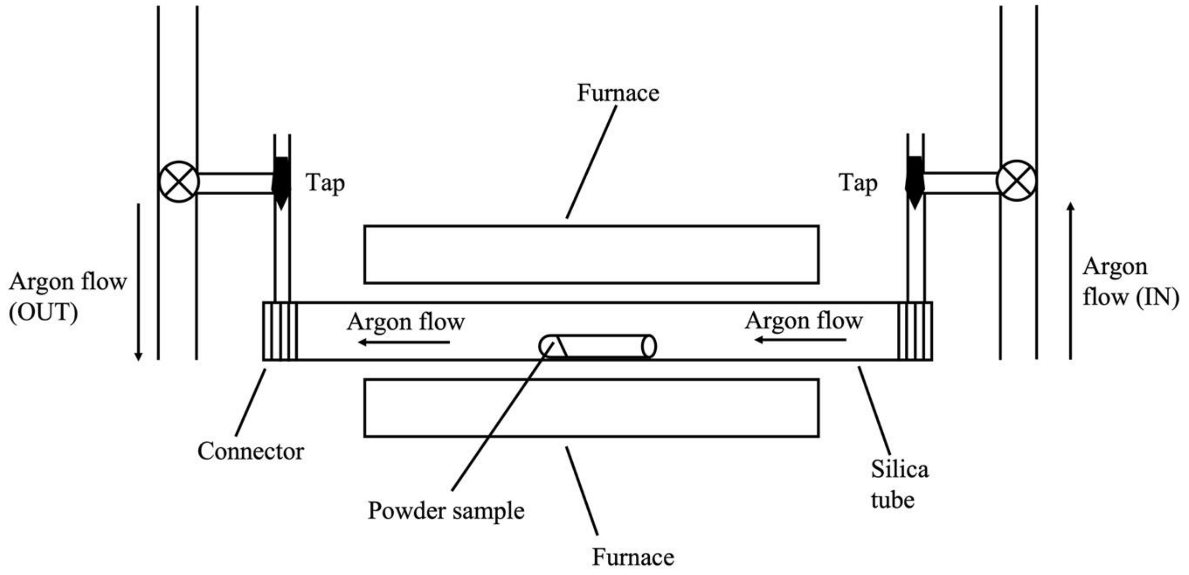

Figure S8. Schematic diagram of the experimental setup for the synthesis of Sample C.

7. Thermogravimetric analysis of Sample C.

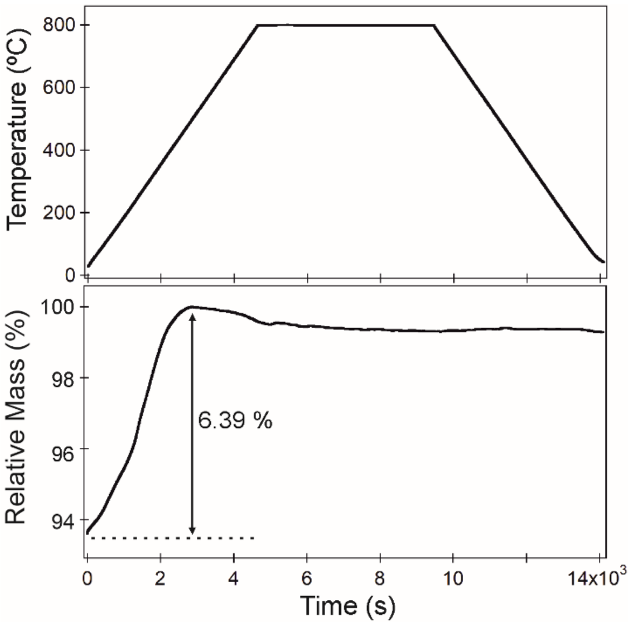

Figure S9. Thermogravimetric data collected while heating Sample C under flowing oxygen.

8. Magnetic measurements in the presence of elemental Co impurities via the 'ferrosubtraction' method.

Procedure used to measure the magnetization of samples containing elemental cobalt: The magnetization of elemental Co is observed to saturate in applied magnetic fields of more than 2 T. Thus the paramagnetic susceptibility of a bulk sample can be measured in the presence of elemental Co impurities by measuring the gradient of magnetization-field isotherms in applied fields larger than 2 T. As shown in Figure S7.

To this end the magnetization of samples was measured in a series of 5 fields between 3 T and 5 T. The magnetization vs. field data were fitted to a linear function, the gradient of which is the paramagnetic susceptibility of the bulk sample and the intercept is the saturated ferromagnetic moment of the sample. Data points with large errors were excluded from fits. All fits had at least 4 data points. This procedure was repeated at 5 K intervals between 5 K and 300 K to measure the temperature dependent susceptibility of samples.

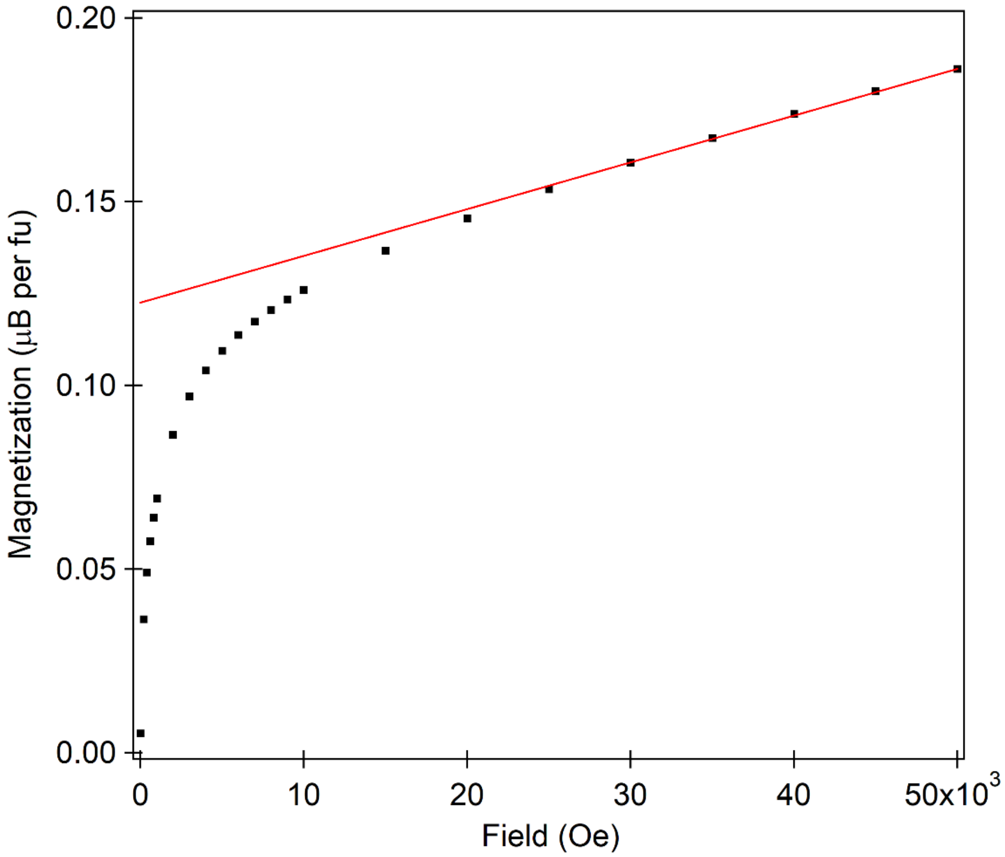

Figure S10. Magnetisation of Sample A measured as a function of applied field at 300 K. A linear fit to high-field region (H > 25000 Oe) yields a gradient which is the paramagnetic susceptibility of the sample, and an intercept which is the saturated ferromagnetic moment of the sample.

9. Magnetic Characterization of Sample A.

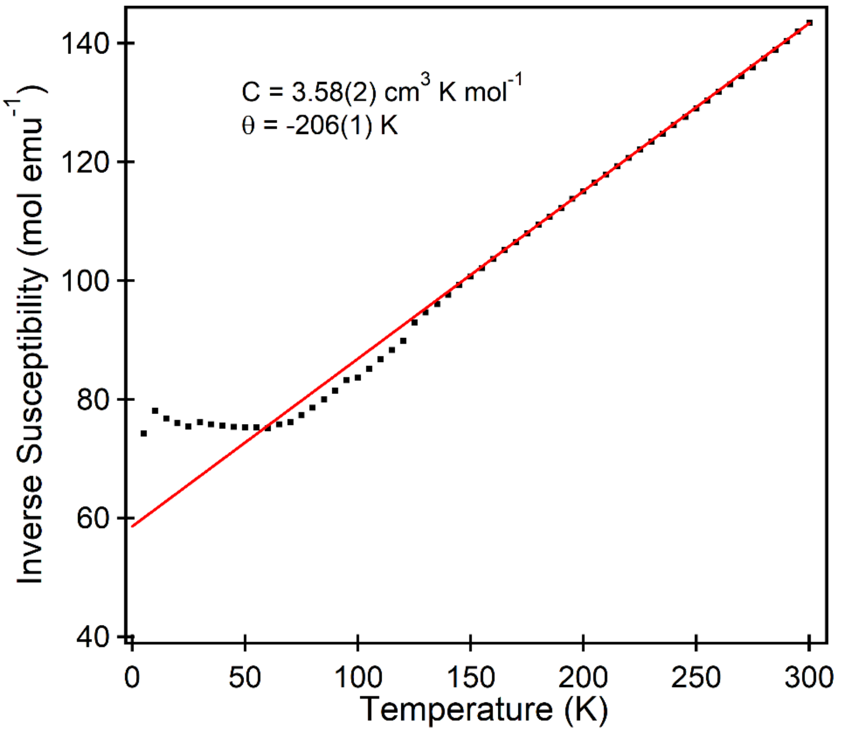

Figure S11. Plot of inverse magnetic susceptibility against temperature for Sample A. Linear fit for T > 150 K, consistent with Curie-Weiss law.

10. Magnetic Characterization of Sample B.

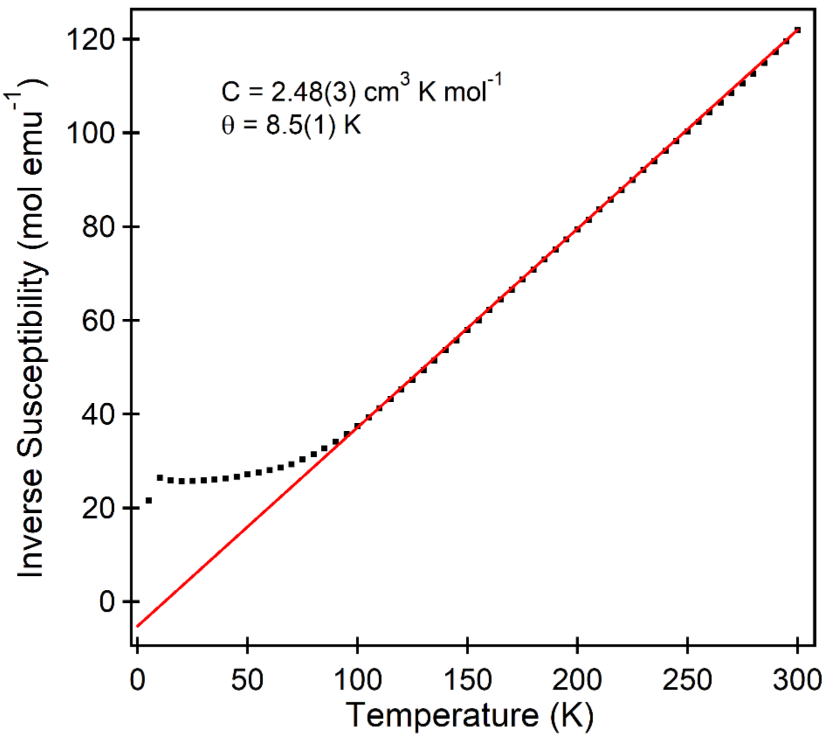

Figure S12. Plot of inverse magnetic susceptibility against temperature for Sample B. Linear fit for T > 150 K, consistent with Curie-Weiss law.

11. Magnetic Characterization of Sample C.

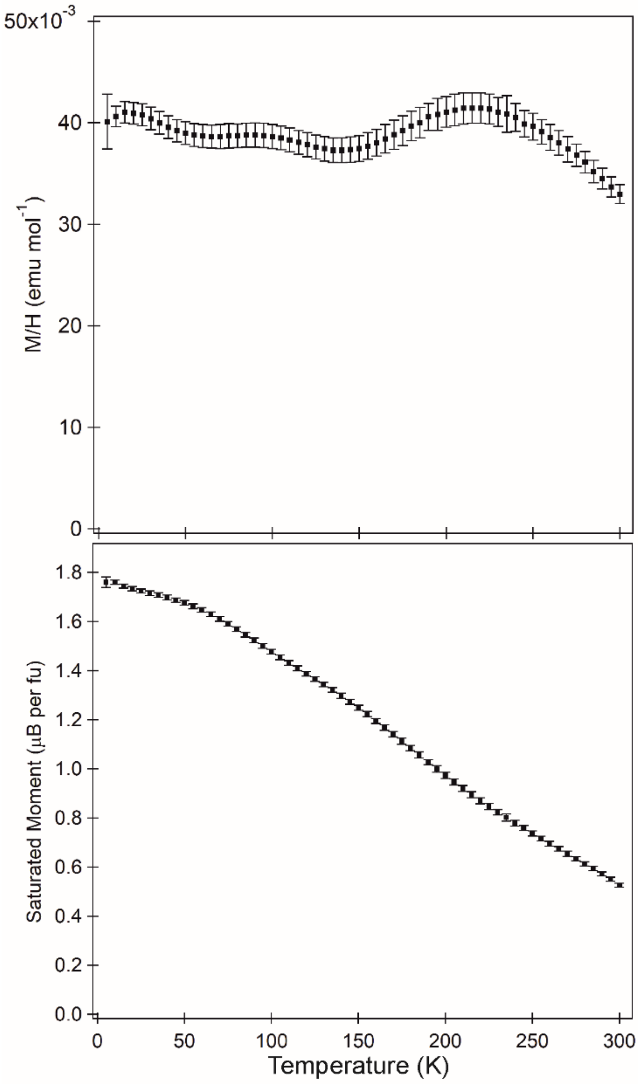

Figure S13. Magnetization data collected from Sample C using the ferrosubtraction procedure.

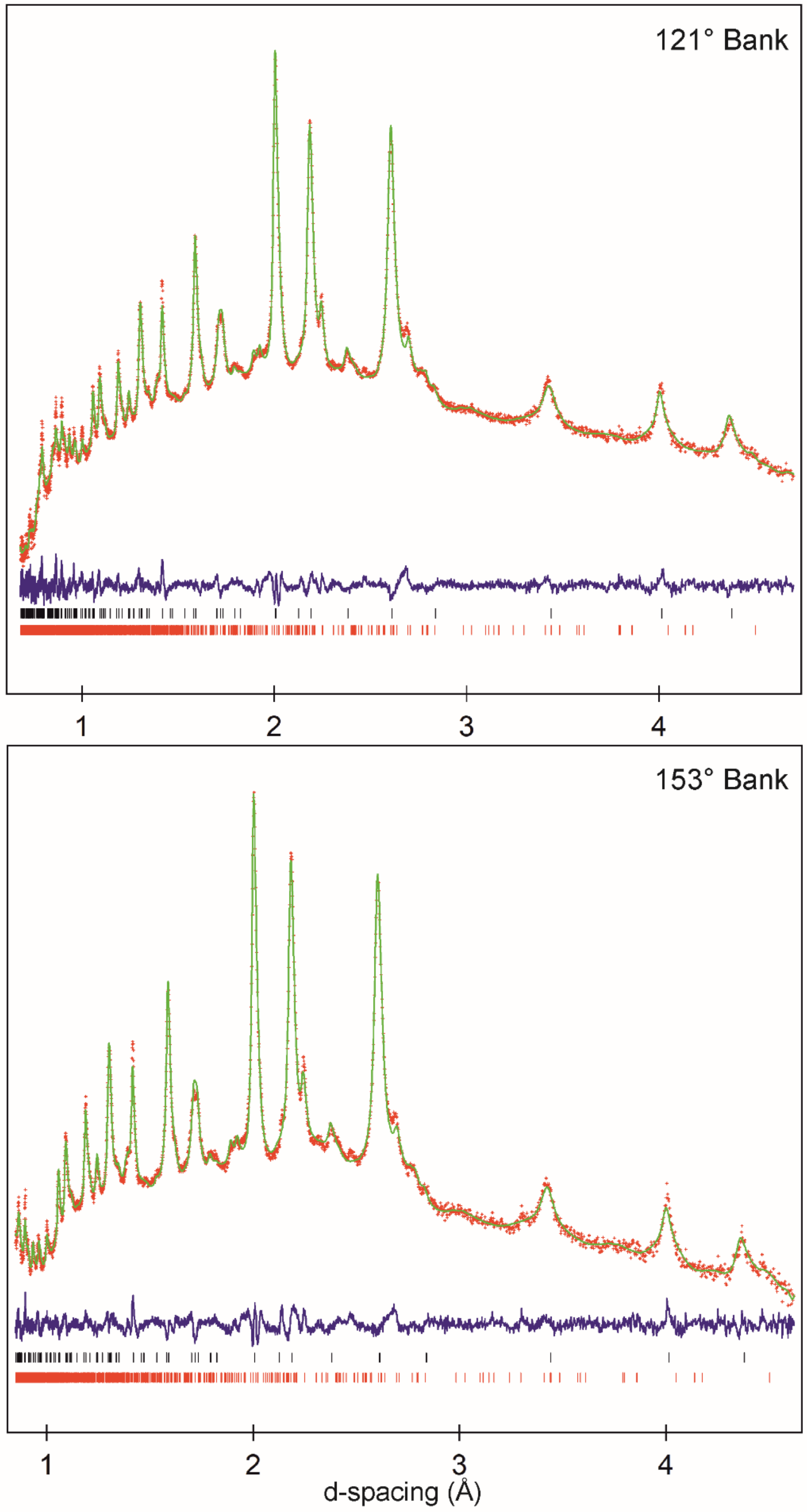

Figure S14. Observed calculated and difference plots from the structural and magnetic refinement of $LaSrCoRuO_4$ against NPD data collected at 5 K. Black tick marks indicate peak positions for $LaSrCoRuO_4$, red ticks $LaSrCoRuO_5$.

Atom	x	y	z	Fraction	B_{iso}	m_x	m_y	m_z
La/Sr	0	$^1/_2$	$^1/_4$	0.5/0.5	0.25(4)			
Co	0	0	0	1	1.86(6)	0	0	1.62(6)
Ru	0	0	$^1/_2$	1	1.86(6)	0	0	0.21(7)
O	0.2516(5)	0.2516(5)	0	1	1.89(5)			
$LaSrCoRuO_4$ – space group *I*4/*mmm* (#139),<br>magnetic space group 139.537 (*I*4/*mm*'*m*')<br>*a* = 5.6689(2) Å, *c* = 6.8578(4) Å, volume = 220.39(2) $Å^3$<br>Formula weight = 450.53 g mol^{-1}, Z = 2<br>weight fraction = 91.7 %								
$LaSrCoRuO_5$ – space group $P112_1$ (#4)<br>*a* = 10.816(9) Å, *b* = 10.825(9) Å, *c* = 8.118(2) Å, *γ* = 91.02(3)°, volume = 950.33(125) $Å^3$<br>Formula weight = 466.53 g mol^{-1}, Z = 8<br>weight fraction = 8.3 %								
Radiation source: Neutron time-of-flight<br>Temperature: 298 K<br>*wRp = 2.04%; Rp = 1.49%*								

Table S2. Parameters from the structural and magnetic refinement of $LaSrCoRuO_4$ against NPD data collected at 5 K.
